# WNT10B Polymorphism in Korean Stroke Patients with Yin Deficiency Pattern

**DOI:** 10.1155/2012/798131

**Published:** 2012-08-08

**Authors:** Mi Mi Ko, Tae-Yong Park, Ji Hye Lim, Min Ho Cha, Myeong Soo Lee

**Affiliations:** Medical Research Division, Korea Institute of Oriental Medicine, 1672 Yuseongdae-ro, Yuseong-gu, Daejeon 305-811, Republic of Korea

## Abstract

WNT10B has been indicated as a potential regulator of adipogenesis *in vivo* and *in vitro* models of obesity. In this study, we analyzed the distribution of WNT10B polymorphism in elderly Korean subjects with cerebral infarction (CI) and Yin Deficiency pattern and Non-Yin Deficiency pattern. A total of 630 CI patients, including 75 with Yin Deficiency pattern and 555 with Non-Yin Deficiency pattern, participated in this study. SNP (G-607C) genotyping was conducted by primer extension using TaqMan probe; five percent of subjects were regenotyped by direct sequencing to confirm the accuracy of the genotyping. The results were analyzed using a multiple logistic regression model to evaluate the genetic association between the G-607C variant and Yin Deficiency pattern. The frequency of the CC genotype of G-607C in the Yin Deficiency pattern group (29.33%) was significantly higher than that in the Non-Yin Deficiency pattern group (23.96%) (*P* = 0.0339 , OR = 2.005 (1.054–3.814)) in a recessive model. This is the first study to demonstrate an association between a WNT10B polymorphism and the Yin Deficiency pattern of traditional Korean medicine (TKM) in a CI patient population. These results suggest that G-607C might be used as a diagnostic genetic marker for Yin Deficiency pattern in stroke patients and in the development of personalized medical care.

## 1. Introduction

Patients with a specific disease exhibit various phenotypes, signs, and symptoms reflecting the cause, nature, and location of the illness, the patient's physical condition, and the patient's treatment. Traditional Korean medicine (TKM), similar to traditional Chinese medicine (TCM), categorizes these phenotypes as patterns, and the procedure for determining the pattern of a particular patient is called “pattern identification” (PI) [1, 2]. Previous reports have described the PI process for differentiating stroke victims with four TKM types: the Fire-Heat pattern, Dampness-Phlegm pattern, Yin Deficiency pattern, and Qi Deficiency pattern [3, 4]. 

The Yin Deficiency pattern indicates a pattern/syndrome resulting from a deficiency of yin fluid and essence, incapable of restraining yang [1]. Furthermore, the Yin Deficiency pattern is typically associated with thinness [5–7]. Conversely, patients with the Qi Deficiency or Dampness-Phlegm patterns tend to be overweight or obese [7, 8].

PI is affected by various environmental factors, such as climate, diet, and lifestyle [9]. However, some reports have shown that inherent factors, such as genetic variations, are correlated with PI [10–14]. For example, a study in Chinese patients with coronary heart disease reported that the frequencies of the *ε*4 allele and *ε*3/4 genotype of the apolipoprotein E gene were significantly higher in patients with the phlegm pattern than in patients with the blood stasis pattern [10]. Recently, we reported that L55 M and C-2033T alleles of the Paraoxonase 1 (PON1) gene, which were correlated with stroke in an East Asian population, were associated with the Dampness-Phlegm pattern among Korean stroke patients [12]. Around the same time, our research group also reported that the C-399T variation of the neuropeptide Y (NPY) gene was associated with the Dampness-Phlegm pattern in Korean stroke patients [13]. However, few genetic polymorphisms have been associated with the Yin Deficiency pattern. 

Wingless-type MMTV integration sites (WNTs) are secreted glycoproteins that function as signaling molecules and are involved in numerous events in human organogenesis, physiology, and pathology [15]. WNT10B, one member of the Wnt family, was first found in human breast carcinoma [16], and several studies have been performed to identify the relationship between WNT10B and human diseases [17]. The Kirikoshi study group found that WNT10B expression was upregulated in various carcinoma cells [18–22], and Christodoulides et al. [23] and Kim et al. [24] reported that WNT10B is negatively correlated with obesity. 

WNT10B is located on chromosome 12q13, and six tightly linked single nucleotide polymorphisms (SNPs) in this gene have been identified in Korean women [24]. Three of these SNPs are in promoter regions: one is in the 5′-untranslated region, and two synonymous SNPs are in exon 5 and 6. Among the three SNPs in the promoter, G-607C has functional activity, modulating the transcriptional activity of WNT10B by altering the binding activity of some transcription factors.

In this study, we analyzed the distribution of G-607C genotypes in elderly Korean subjects with Yin Deficiency pattern, which is clinically different from other patterns.

## 2. Materials and Methods 

### 2.1. Study Subjects

The data for this analysis were collected as parts of the “The Fundamental Study for the Standardization and Objectification of Pattern Identification in TKM for Stroke (SOPI-Stroke)” project of the Korean Institute of Oriental Medicine (KIOM) [3, 4]. 

The study group was composed of patients with cerebral infarction (CI) who were admitted to one of the thirteen Korean oriental medical hospitals participating in this study from November 2006 to March 2011. To be eligible for the study, participants had to be diagnosed with stroke within 30 days of symptom onset as confirmed by imaging, such as computerized tomography (CT) or magnetic resonance imaging (MRI). Patients with traumatic stroke, such as subarachnoid, subdural, or epidural hemorrhage, were excluded from this study. 

 After obtaining written informed consent from all subjects, the data were collected using a case report form (CRF), including general subject characteristics, such as the diagnosis, medical history, and score on the Korean standard pattern identification for stroke (K-SPI-Stoke), which was developed by an expert committee organized by the KIOM [3, 4]. The PI diagnosis of each patient was made by two expert TKM doctors, and subjects who received different diagnoses from the two doctors were excluded. Subjects for whom BMI, waist circumference, or other physical data were missing were also excluded. A total of 630 CI patients were classified as having a Yin Deficiency pattern (*N* = 75) or Non-Yin Deficiency pattern (*N* = 555). The general characteristics of Non-Yin Deficiency pattern patients are shown in Supplemental Table  1 (see Supplementary Material available online at doi:10.1155/2012/798131). This study was approved by the Institutional Review Boards of the KIOM and the Oriental Medical Hospitals. 

### 2.2. Preparation of Genomic DNA and Identification of SNP

**Figure 1:**
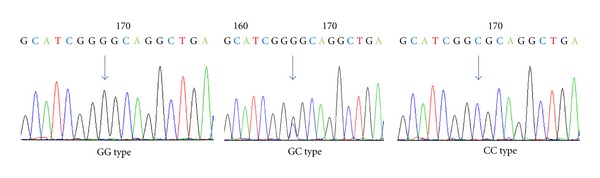
Polymorphic sites identified in the WNT10B. G-607C SNP is indicated by arrows.

 Genomic DNA from each subject was extracted from whole blood using a GeneAll Genomic Isolation Kit (GeneAll, Seoul, Korea). The WNT10B G-607C polymorphism of each subject was genotyped using the TaQman method with polymerase chain reaction (PCR) primers and TaQman probes purchased from ABI Inc. (Applied Biosystems Inc., USA) according to the manufacturer's protocol. Additionally, the genotypes of five percent of the participants were redetermined by direct sequencing to verify the accuracy of the TaQman method (Figure 1). The primers used for the amplification and genotyping of G-607C variation are described in Supplemental Table 2. The genotyping error rate of the TaQman method in this study was 0.3% based on the direct sequencing of PCR products (data not shown), and the Kappa value was 0.98, demonstrating good accuracy. Hardy-Weinberg equilibrium (HWE) was evaluated by chi-square test using Haploview 4.2 (http://www.genenames.org/). 

### 2.3. Statistical Analysis

Data were statistically analyzed with SAS software, version 9.1.3 (SAS Institute Inc., Cary, NC). All continuous variables were subjected to a Kolmogorov-Smirnov normality test. Differences in continuous variables were determined using parametric (Student's *t*-test) or nonparametric (Wilcoxon rank-sum test) tests. Categorical variables were compared with a chi-square test or Fisher's exact test.

The association of the SNP with the Yin Deficiency pattern versus Non-Yin Deficiency pattern was performed by multiple logistic regression adjusted for age, body mass index (BMI), waist-hip ratio (WHR), and triglyceride levels, and odds ratios (ORs) with 95% confidence intervals (95% CI) were calculated. To investigate whether the G-607C polymorphism is associated with the clinical parameters of the study subjects, we performed a statistical analysis using a general linear model adjusted for sex, age, smoking, and drinking status. Statistical significance was set at *P* < 0.05.

## 3. Result

The general characteristics of CI patients classified as Yin Deficiency pattern or Non-Yin Deficiency pattern according to the PI of TKM are shown in Table 1. The mean age of the Yin Deficiency pattern patients was significantly higher than that of the Non-Yin Deficiency pattern patients (*P* = 0.002). Additionally, triglyceride levels were significantly lower in the Yin Deficiency pattern group than in the Non- Yin Deficiency pattern group (*P* = 0.0009). 

The clinical differences in the body characteristics between Yin Deficiency and Non-Yin Deficiency patterns are shown in Table 2. The mean weight, BMI and waist circumference of the Yin Deficiency pattern patients, was significantly lower than that of the Non-Yin Deficiency pattern subjects after adjustment for age and triglyceride levels (*P* = 0.0002, *P* = 0.0020 and *P* < 0.0001, resp.).

The G-607C SNP in WNT10B was in HWE (*P* = 0.396) according to the International HapMap Project guidelines, and the minor allele frequency (MAF) in this study was 0.495, which is slightly higher than that observed in previous studies among Korean females and Caucasians in USA [24, 25]. 

Table 3 shows the G-607C SNP distribution in the Non-Yin Deficiency and Yin Deficiency patients. The ratio of subjects with the CC genotype in the Yin Deficiency pattern group (29.33%) is smaller than that in the Non-Yin Deficiency pattern group (23.96%), after adjustment for age, BMI, WHR, and triglyceride levels (*P* = 0.0339, OR = 2.005 (1.054–3.814)). The frequency of the C allele in Yin Deficiency pattern patients is 50.0%, slightly higher than that in the Non-Yin Deficiency pattern patients (49.46%), although this difference was not significant. Additionally, the frequency of the G allele in the Yin Deficiency pattern group (70.67%) is smaller than that in the Non-Yin Deficiency pattern group (74.95%), but this difference was not statistically significant. 

Table 4 shows the comparison of obesity phenotypes and serum parameters according to G-607C genotype. The mean level of total cholesterol tended to decrease in subjects with the CC genotype compared with the GG or GC genotype in the recessive model (*P* = 0.0634). 

## 4. Discussion

PI is a traditional diagnosis system developed over several hundred years in East Asia [1, 2], but there remains a lack of scientific evidence supporting its use due to its high dependence on subjective diagnostic indicators. Recently, some clinical differences among patterns were reported, and genetic factors have been correlated to PI in certain diseases [10–14].

WNTs are the ligands of Frizzled receptors and are involved in many physiological pathways by regulating the Wnt/*β*-catenin signaling pathway [26]. Among WNT family genes, WNT10B is expressed in carcinoma cells [18–22] and adipocytes [27, 28] and is known to inhibit adipogenesis. Ross et al. showed that the overexpression of WNT10B in 3T3L1 preadipocytes inhibited adipogenesis by inhibiting C/EBP*α* and PPAR*γ*2, transcription factors that accelerate adipogenesis [27], and Bennett et al. also reported that WNT10B inhibits adipogenesis by inhibiting glycogen synthase kinase 3 activity [28]. Another study also showed that transgenic mice with WNT10B driven by the FABP4 promoter have 50–70% less adipose tissue weight than ob/ob mice [29]. Recently, G-607C SNP, which is located in a promoter region of WNT10B, was found to alter the expression of WNT10B and was significantly associated with a decrease in abdominal fat area in Korean female subjects [24].

TKM categorizes stroke patients according to their related internal disease symptoms. Previously, Lee et al. described PI for four patterns in stroke patients (Fire-Heat pattern, Dampness-Phlegm pattern, Yin Deficiency pattern, and Qi Deficiency pattern) as part of the “SOPI-Stroke” project of the KIOM [3, 4]. The Yin Deficiency pattern in stroke patients results from a deficiency of yin with diminished moistening and inability to restrain yang, which is usually manifested as emaciation, dizziness, tinnitus, dryness of the mouth and throat, constipation, dark urine, afternoon fever, malar flush, night sweats, reddened tongue with scanty coating, and rapid fine pulse [1–4]. Moreover, patients with Yin Deficiency pattern are nonobese and gaunt [1, 5, 8]. Wu et al. reported that Pro12Ala SNP of PPARG polymorphism were associated with Yin Deficiency pattern in a Chinese Han population [14]. However, no genetic factors associated with the Yin Deficiency pattern have yet been established in a Korean population. 

In this study, we analyzed the association of the G-607C SNP of WNT10B with a Yin Deficiency pattern in elderly Korean subjects with CI.

The Yin Deficiency pattern was related with decreased obesity. In this study population, the BMI and waist circumference in the Yin Deficiency group were significantly lower than that in the Non-Yin Deficiency group, and WHR was also slightly lower in subjects with Yin Deficiency (Table 2). This result was similar to those of other studies [7, 8]. Zhu et al. showed that frequency of overweight in subjects with Yin Deficiency was slightly lower than that in the overall study population [7], and Yin Deficiency patients with type 2 diabetes mellitus were usually non-obese [8]. 

In this study, the frequency of subjects with the CC genotype in the G-607C SNP of WNT10B was 29.33% in the Yin Deficiency group, significantly higher than that in the Non-Yin Deficiency group (23.96%) (OR = 2.005) in the recessive model (Table 3). We also confirmed that the G-607C SNP was associated with a trend toward decreased serum lipids and total cholesterol (Table 4). These data showed that correlation between the G-607C SNP and Yin Deficiency in elderly Korean subjects with CI might be related to a decrease in obesity indices. This result suggests that PI may be affected not only by environmental factors but also by inherent factors such as genetic variations.

 This study showed, for the first time, that WNT10B polymorphism is associated with Yin Deficiency pattern in a CI patient population. Thus, this SNP can be used as a diagnostic genetic marker for Yin Deficiency pattern in stroke patients and in the development of personalized medical care. However, this study included several limitations. First, this is a simple cross-sectional study, not a longitudinal study. Second, this study does not have a sufficient sample size to generalize the relationship between WNT10B and Yin Deficiency. Further studies should be performed in subjects of other ethnicities to generalize the conclusions of this study.

## Supplementary Material

This summary is the general characteristics of Non-Yin Deficiency pattern patients, including 100 with QD pattern, 264 with DP pattern and 161 with FH pattern.

## Figures and Tables

**Table 1:** Demographic parameters of study subjects.

Characteristics	Non-YD	YD	*P*
*N*	555	75	
Sex (M/F)	229/326	32/43	0.8166
Age (year)	68.38 ± 10.44	73.27 ± 10.44	**0.000** **2**
Smoking (none/stop/active)	314/103/138	47/17/11	0.1410
Drinking (none/stop/active)	295/71/189	47/10/18	0.2071
TOAST classification			
LAA	161	27	0.4005
CE	40	4	
SVO	322	37	
SOE	12	3	
SUE	18	4	
Medical history			
TIA (*n*, %)	69 (12.52)	11 (14.86)	0.5712
Hypertension (*n*, %)	339 (61.08)	49 (65.33)	0.4773
Hyperlipidemia (*n*, %)	79 (14.34)	8 (10.67)	0.3886
Diabetes (*n*, %)	147 (26.58)	17 (22.67)	0.4688
Heart disease (*n*, %)	36 (6.51)	4 (5.33)	>0.999
Serum parameters			
GOP (U/mL)	26.75 ± 14.72	26.95 ± 14.03	0.9134
GPT (U/mL)	24.80 ± 18.38	24.64 ± 18.86	0.9439
Total cholesterol (mg/dL)	188.41 ± 48.97	184.44 ± 44.66	0.5060
Triglyceride (mg/dL)	165.19 ± 127.14	133.57 ± 64.66	**0.00** **09**
HDL-cholesterol (mg/dL)	43.23 ± 11.10	43.47 ± 11.77	0.8662
FBS (mg/dL)	113.06 ± 42.26	121.52 ± 50.20	0.1487

Data were expressed as frequencies for categorical variables and as the mean ± standard deviation for continuous variables. YD: Yin Deficiency. TOAST: Trial of ORG 10172 in acute stroke treatment. LAA: large-artery atherosclerosis. CE: cardioembolism. SVO: small-vessel occlusion. SOE: stroke of other etiology. SUE: stroke of undetermined etiology. TIA: transient ischemic attack. *P* value was calculated using the Student's *t*-test or Wilcoxon rank-sum test for continuous variables and the chi-square test or Fisher's exact test for categorical variables. *P* values with statistical significance are shown in bold (<0.05).

**Table 2:** Difference in body characteristics among study subjects.

Characteristics	Non-YD	YD	*P*
*N*	555	75	
Weight (kg)	62.76 ± 10.90	56.03 ± 11.34	**0.0002**
BMI (kg/m^2^)	24.41 ± 3.41	22.70 ± 3.58	**0.00** **20**
Waist circumference (cm)	88.79 ± 9.11	82.20 ± 9.92	**<** **0** **.0001**
WHR	0.95 ± 0.12	0.95 ± 0.22	0.7839

All results are expressed as the mean ± standard deviation. BMI: body mass index. WHR: waist-hip ratio. *P* values: adjusted for age and triglyceride levels using a general linear model. *P* values with statistical significance are shown in bold (<0.05).

**Table 3:** Genotype distribution of the G-607C polymorphism in patients with Yin Deficiency pattern versus non-Yin Deficiency pattern.

Model	Genotype	Non-YD	YD	^†^OR [95% CI]	*P*
Allele	G	561 (50.54)	75 (50.0)	1.262 [0.818, 1.946]	0.2927
	C	549 (49.46)	75 (50.0)		
^§^Do	GG	139 (25.05)	22 (29.33)	0.850 [0.422, 1.712]	0.6495
	GC + CC	416 (74.95)	53 (70.67)		
^§^R	GG + GC	422 (76.04)	53 (70.67)	2.005 [1.054, 3.814]	**0.0** **339**
	CC	133 (23.96)	22 (29.33)		

The data are presented as frequencies (percentages). ^†^ORs after adjustment for age, BMI, WHR, and triglyceride levels. *P* values were calculated by logistic regression analysis. ^§^Do and R denote dominant and recessive models, respectively. *P* values with statistical significance are shown in bold (<0.05).

**Table 4:** Association of G-607C polymorphism with clinical parameters among study subjects.

Variable	Genotype			*P*		
	GG (*n* = 161)	GC (*n* = 314)	CC (*n* = 155)	^§^Co	^§^Do	^§^R
Body characteristics						
Weight (kg)	61.44 ± 11.91	62.74 ± 11.06	60.89 ± 10.50	0.2681	0.7136	0.1711
BMI (kg/m^2^)	24.23 ± 3.44	24.32 ± 3.63	24.01 ± 3.17	0.7647	0.8895	0.4686
Waist circumference (cm)	87.86 ± 9.91	88.37 ± 9.67	87.13 ± 8.52	0.5062	0.9268	0.2835
WHR	0.95 ± 0.10	0.95 ± 0.12	0.95 ± 0.18	0.9547	0.9179	0.7606
Serum parameters						
GOP (U/mL)	24.89 ± 10.78	27.29 ± 15.73	27.70 ± 15.66	0.1623	0.0602	0.3483
GPT (U/mL)	23.73 ± 16.78	25.25 ± 19.39	24.92 ± 18.10	0.7313	0.4291	0.8164
Total cholesterol (mg/dL)	191.67 ± 52.29	189.18 ± 48.42	181.55 ± 43.91	0.1521	0.2479	0.0634
Triglyceride (mg/dL)	171.75 ± 150.35	161.46 ± 116.84	151.08 ± 96.69	0.3631	0.2243	0.2685
HDL-cholesterol (mg/dL)	43.79 ± 11.15	43.40 ± 11.09	42.41 ± 11.40	0.5582	0.5173	0.3029
FBS (mg/dL)	108.86 ± 29.41	117.44 ± 51.19	112.46 ± 36.74	0.1569	0.1072	0.6417

The data are presented as the mean ± standard deviation. *P* values: adjusted for sex, age, smoking, and drinking status using a general linear model. ^§^Co, Do and R denote codominant, dominant, and recessive models, respectively.
