# Supplementary material for: WNT10B Polymorphism in Korean Stroke Patients with Yin Deficiency Pattern
Source: Evid Based Complement Alternat Med. 2012 Aug 8;2012:798131. doi: 10.1155/2012/798131 (PMC3426177; doi:10.1155/2012/798131)
Supplement: Supplementary file 1 — This summary is the general characteristics of Non-Yin Deficiency pattern patients, including 100 with QD pattern, 264 with DP pattern and 161 with FH pattern. [file 798131.f1.docx]

Supplemental Table 1. Demographic parameters of Non- Yin Deficiency pattern patients

| Characteristics | QD | DP | FH |
| --- | --- | --- | --- |
| N | 100 | 264 | 161 |
| sex (M/F) | 49/51 | 117/147 | 63/128 |
| age (year) | 68.90±11.65 | 67.90±9.71 | 68.77±10.75 |
| smoking (none/stop/active) | 73/14/13 | 161/43/60 | 80/46/65 |
| drinking (none/stop/active) | 66/7/27 | 151/35/78 | 78/29/84 |
| weight (kg) | 57.39±9.75 | 63.74±10.28 | 64.37±11.51 |
| BMI (kg/m^2^) | 23.44±3.51 | 24.90±3.24 | 24.24±3.48 |
| waist circumference (cm) | 86.50±8.54 | 90.45±9.02 | 87.60±9.17 |
| WHR | 0.94±0.15 | 0.95±0.10 | 0.95±0.13 |
| *TOAST classification* |  |  |  |
| LAA | 19 | 67 | 75 |
| CE | 11 | 14 | 15 |
| SVO | 64 | 166 | 92 |
| SOE | 2 | 8 | 2 |
| SUE | 3 | 9 | 6 |
| *medical history* |  |  |  |
| TIA (n, %) | 13 (13.0) | 32 (12.17) | 24 (12.77) |
| hypertension (n, %) | 52 (52.0) | 166 (62.88) | 121 (63.35) |
| hyperlipidemia (n, %) | 10 (10.1) | 42 (15.91) | 27 (14.36) |
| diabetes (n, %) | 30 (30.0) | 69 (26.24) | 48 (25.26) |
| heart disease (n, %) | 7 (7.0) | 20 (7.6) | 9 (4.74) |
| *serum parameters* |  |  |  |
| GOP (U/ml) | 26.47±17.73 | 25.58±11.47 | 28.51±16.75 |
| GPT (U/ml) | 25.80±24.70 | 23.07±13.89 | 26.66±19.79 |
| total cholesterol (mg/dL) | 188.10±47.93 | 193.55±52.30 | 181.47±43.88 |
| triglyceride (mg/dL) | 164.11±151.21 | 179.63±139.10 | 145.48±87.40 |
| HDL-cholesterol (mg/dL) | 44.13±11.88 | 43.95±11.03 | 41.74±10.72 |
| FBS (mg/dL) | 116.0±62.65 | 113.50±37.62 | 110.91±35.84 |

Data were expressed as frequencies for categorical variables and expressed as the mean ± standard deviation for continuous variables. YD: Yin Deficiency; QD: Qi Deficiency; DP: Dampness-phlegm; FH: Fire-heat.

Supplemental Table 2. Primer sets for PCR amplification and G-607C genotyping

| SNP | Direction | PCR amplification primer | Probe | Genotyping primer | Sequencing primer |
| --- | --- | --- | --- | --- | --- |
| G-607C | Forward | C1125211_10_F | VIC | C1125211_10_V | CCAAGACCAGGCTCTTTCC |
|  | Reverse | C1125211_10_R | FAM | C1125211_10_F | CTTCTCGGTGTGGGACAGT |
